# A superior loading control for the cellular thermal shift assay

**DOI:** 10.1038/s41598-022-10653-7

**Published:** 2022-04-23

**Authors:** Alexandré Delport, Raymond Hewer

**Affiliations:** Discipline of Biochemistry, School of Life Sciences, University of KwaZulu-Natal, Pietermaritzburg, 3201 South Africa

**Keywords:** Biochemistry, Drug discovery

## Abstract

The cellular thermal shift assay (CETSA), as a method to determine protein–ligand interaction and cellular protein modification, has rapidly become routine laboratory practice. However, current options to determine that (1) sample was loaded in each lane of the analysed western blot and (2) the amount loaded was equal, are suboptimal. Here, we report that the αC-terminal fragment of the amyloid precursor protein (APP-αCTF), detected in several wild-type mammalian cell lines, is a highly stable, soluble protein equally present from 4 to 95 °C. We demonstrate that the level of traditional loading controls (vinculin, GAPDH, β-actin, heat-shock chaperone 70 and superoxide dismutase-1) are all temperature sensitive. Additionally, both APP-CTFs (α and β) behaved similarly upon temperature exposure while APP-βCTF levels were not influenced by the presence of a binding ligand either. This emphasises that these proteins can be used as a loading control in the unlikely event of off-target binding during ligand screening. A working example is also presented for mitogen-activated protein kinase kinase in the presence of two inhibitors, PD184352 and U0126, where APP-αCTF was used to normalise the data across experimental replicates. A reduction in data variance and standard deviations was observed after normalisation. Conclusively, APP-αCTF is a superior CETSA loading control that can be used as a standard for this technique.

## Introduction

The cellular thermal shift assay (CETSA) has become a popular method to determine a change in protein stability in a cellular environment, with over 160 experimental articles describing this technique having been published following its introduction in 2013^1^. The assay is based on the principle that a protein will unfold and aggregate when exposed to increasing temperature, thereby moving from a soluble to an insoluble state^2^. By quantifying the amount of remaining soluble protein at each exposed temperature, distinct melting curves can be generated and a point where 50% of the protein remains soluble—the aggregation temperature (Tagg) (or sometimes melting temperature (Tm))—can be determined^2^. When a protein is stabilised, for example in the presence of a binding ligand^3^, through protein modifications^4^ or protein–protein interactions^5,6^, a discernible shift (generally an increase) in the protein Tagg is observed. For protein–ligand binding in particular, this assay provides unique advantages over other thermal shift assays as engagement occurs intracellularly where additional pharmacological properties (such as ligand permeability and toxicity) may also be inferred.

The CETSA essentially detects a fluctuation of a target protein in the soluble form—a change in protein concentration—and is most easily done by performing quantitative western blot (qWB) analyses on the soluble protein fractions after temperature exposure^1,3^. Fluctuations in protein level can be brought about through protein stabilisation but may also arise, or be contrived, through unequal sample loading. To provide certainty that detected shifts in protein aggregation temperature arise through authentic protein stabilisation and not sample loading errors, it is necessary to show that (1) sample was loaded in each lane of the analysed qWB and (2) that the amount loaded was equal. Traditional qWB loading controls have been utilised previously but are ill-suited for this unintended purpose. Vinculin, β-actin, GAPDH, α-tubulin and even heat-shock chaperone 70 (HSP70) decrease in concentration as temperature increases^7–11^. Superoxide dismutase-1 (SOD-1), a promising candidate owing to its reported stable range of 35–95 °C^12–14^, was found to transition through different unfolded states with increasing temperature^13,15^. Therefore, the identification of a temperature insensitive protein—one which remains stable over a broad range of temperatures and that can be detected in commonly used cell lines—is required as a more appropriate control for this assay. Understandably, a target for which antibodies are easily attainable would be advantageous.

The amyloid precursor protein (APP), a ubiquitously expressed mammalian transmembrane protein, can be sequentially cleaved by several secretases including α-, β-, δ- and γ-secretases, and others, to produce an array of smaller APP fragments including the soluble APPs, C-terminal fragments (CTFs) and smaller peptides (e.g., P3, Aβ etc.)^16,17^. The cleavage of full-length APP by α- or β-secretase produces the αCTF (C83, ~ 12 kDa) or βCTF (C99, ~ 14 kDa), respectively^16,17^. Under normal conditions, full-length APP is preferentially cleaved by α-secretase favouring APP-αCTF formation over APP-βCTF^16^. Therefore, αCTF can be readily detected in mammalian cell lines^18,19^.

Previously, we used the CETSA to establish the Tagg of full-length APP at 56.7 ± 0.7 °C, where it was also noted that the solubility of the APP-CTFs, simultaneously detected with the antibody used, was unaffected by temperature exposure^20^. Here, we further probe this phenomenon and demonstrate how these APP-CTFs represent a more appropriate loading control for the CETSA. Specifically, APP-αCTF, which was readily detected in five cell lines, was found to be a stable soluble protein with constant detection between 4 and 95 °C. Moreover, both APP-CTFs (α- and β) behaved similarly upon temperature exposure while APP-βCTF levels were not influenced by the presence of a binding ligand either. Other commonly used loading controls, vinculin, β-actin, GAPDH, HSP70 and SOD-1, all exhibited temperature sensitivities in our study, as previously reported^7–11^. By means of example, we validated the use of APP-αCTF as a CETSA loading control for the kinase, MEK (mitogen-activated protein kinase kinase), in the presence of two inhibitors using the wild-type HEK293 cell line.

## Results

**Figure 1:**
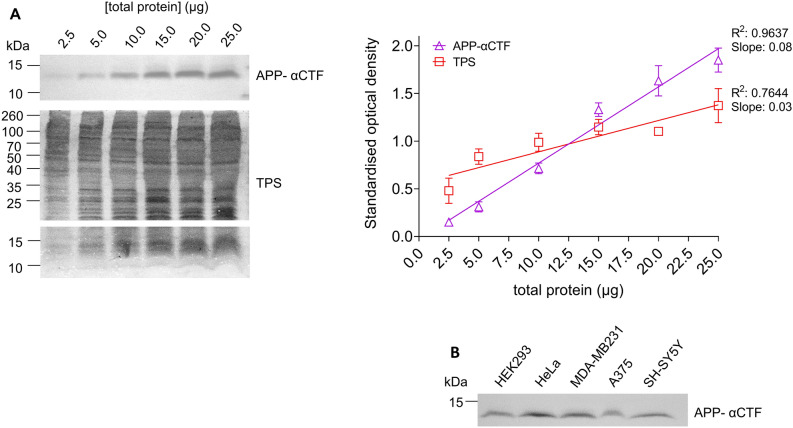
The detection of APP-αCTF in relation to protein concentration, and in five common mammalian cell lines. (**A**) Western blot analysis of HEK293 cell lysate with APP-αCTF detection as total protein concentration increases (2.5–25 µg) as compared to amido black total protein stain (TPS) with corresponding standardised optical density plots as analysed for APP-αCTF (open purple triangle) and TPS (open red square) as mean ± SD showing R^2^ and slope for each (n = 3). (**B**) Western blot analysis of cell lysates from HEK293, Hela, MDA-MB-231, A375 and SH-SY5Y cell lines showing detection of APP-αCTF (10 µg total protein loaded/well). Cropped western blots are shown—see supplementary information file, raw data images for full-length western blots.

Prior to assessing the suitability of APP-αCTF as a thermally stable loading control for the CETSA, we sought to ensure that detection intensity of this protein was directly proportional to total protein concentration in a cell lysate. Here, the level of APP-αCTF was found to increase linearly with total protein concentration, in a manner comparable to the amido black total protein stain (Fig. 1A). Moreover, no saturation by the anti-APP_CTF antibody (1:1500 dilution) was observed up to 25 µg of loaded protein as confirmed by linear regression (R^2^ = 0.9637) (Fig. 1A). We further demonstrated that APP-αCTF was readily detected in five commonly used cell lines of various human origin, HEK293, HeLa, MDA-MB231, A375 and SH-SY5Y, at 10 µg of total protein (Fig. 1B). Our results accompany previous research that describes the detection of APP-αCTF in cell lysates from PC12, KNS-42, HT22, H4 and CHO cell lines as well^18,19,21–23^. Moreover, proteomic analysis of eleven common cell lines (A549, GAMG, HEK293, Hela, HepG2, Jurkat, K562, LnCap, MCF-7, RKO, U2OS) showed the presence of APP (except in the K562 cell line while the Jurkat cell line was inconclusive) and ADAM10 (one of the α-secretase suspected to cleave APP^16,17^, in all)^24^ suggesting APP-αCTF could be present in these cases as well. Therefore, APP-αCTF appears to be ubiquitously expressed in several mammalian cell lines. Overall, the level of APP-αCTF and the sensitivity of the anti-APP_C99 antibody were suitable to detect changes in protein level as is required for a protein loading control.

**Figure 2:**
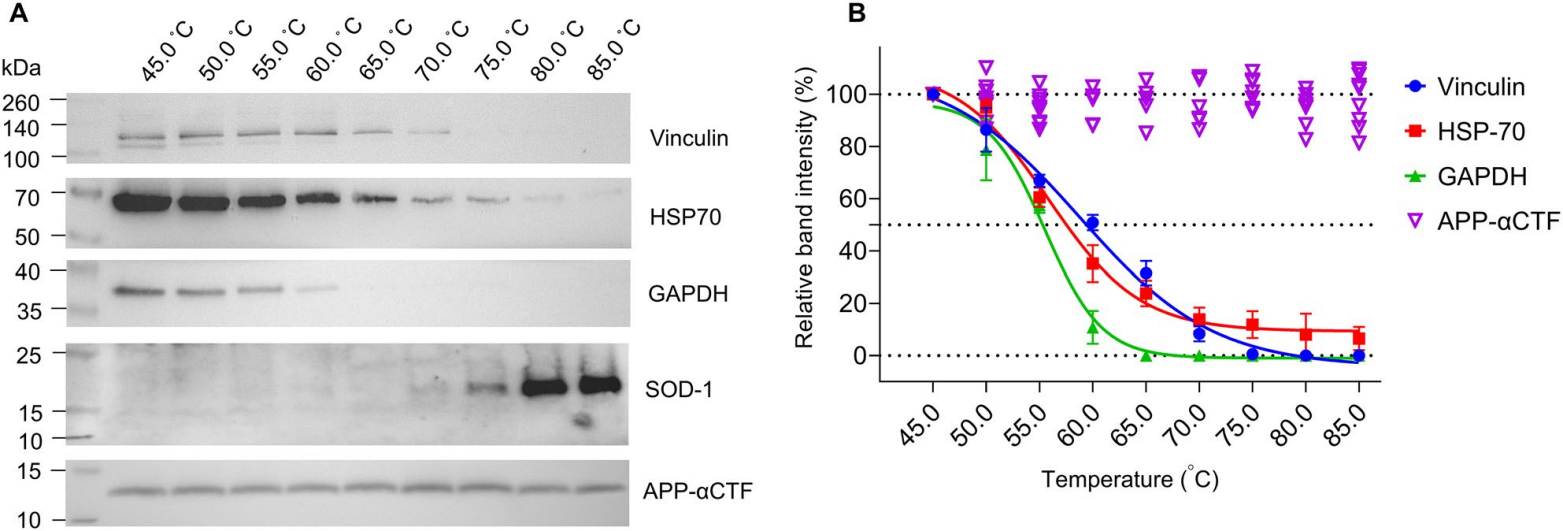
Cellular protein stability, over a 45–85 °C temperature range, of two commonly used protein loading controls (Vinculin (filled blue circle) and GAPDH (filled green triangle)) and temperature stable proteins, heat shock protein 70 (HSP70 (filled red square)) and superoxide dismutase-1 (SOD-1) in comparison to APP-αCTF (open purple inverted triangle). (**A**) A representative cropped western blot of each analysed protein in the soluble fraction from HEK293 cell lysate after temperature exposure with (**B**) the corresponding relative band intensity sigmoidal curves as mean ± SD used to determine the Tagg for each, calculated by setting the highest intensity for each data set as 100% and the curve generated using the Boltzmann sigmoidal equation (n ≥ 3). Also refer to Figs. S1 and S2 (see supplementary information file, raw data images for full-length western blots).

**Figure 3:**
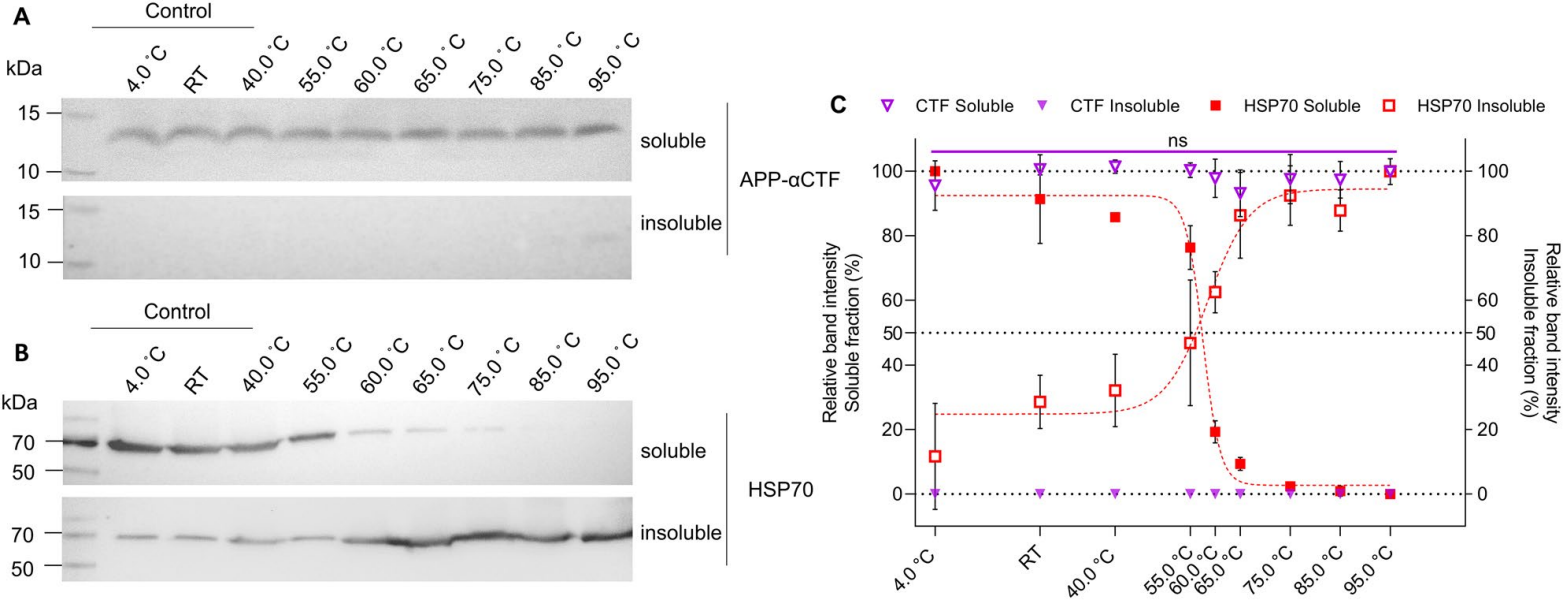
The detection of soluble and insoluble APP-αCTF and HSP70, including 4.0 °C and room temperature (RT) controls. (**A**) A representative cropped western blot of APP-αCTF in the soluble (open purple inverted triangle) and insoluble fraction (filled purple inverted triangle) from HEK293 cell lysate after temperature exposure (4 °C, RT, 40–95 °C) and (**B**) a representative western blot of HSP70 in the soluble (filled red square) and insoluble (open red square) fraction from HEK293 cell lysate after temperature exposure (4 °C, RT, 40–95 °C) with (**C**) the corresponding relative band intensity sigmoidal curves, for both soluble (left y-axis) and insoluble (right y-axis) protein, as mean ± SD calculated by setting the highest intensity for each data set as 100% and each curve generated using the Boltzmann sigmoidal equation (n = 3). Multiple paired t-test was performed for soluble APP-αCTF (purple line) where ns, not significant. See supplementary information file, raw data images for full-length western blots.

We subsequently evaluated the thermal stability of APP-αCTF over a broad temperature range (4 °C, room temperature (RT) and 40–95 °C) and as compared to the other commonly used loading controls—vinculin, HSP70, GAPDH, SOD-1 and β-actin (Figs. 2, 3, Supplementary Figs. S1 and S2). APP-αCTF protein level remained constant up to 95 °C (including 4 °C and RT controls) yielding no significant difference across all temperatures assessed (*p* > 0.05) (Fig. 3C). Importantly, the APP-βCTF, produced only by overexpressing APP^20^, also yielded constant protein levels up to 85 °C (Supplementary Fig. S2). No significant difference was achieved when αCTF and βCTF were directly compared, suggesting the two APP-CTF fragments behave similarly within the CETSA (*p* > 0.05) (Supplementary Fig. S2). In contrast to APP-CTFs, the other loading control proteins displayed temperature sensitivities. Specifically, no discernible detections were observed for vinculin at > 70.0 °C, GAPDH at > 60.0 °C and β-actin at > 50.0 °C (with Taggs of 58.4 ± 1.0 °C and 55.2 ± 0.4 °C calculated for vinculin and GAPDH, respectively) (Fig. 2 and Supplementary Fig. S1). Moreover, the level of HSP70 also decreased with increasing temperature, although protein was detected up to 85 °C (Figs. 2A and 3). The Tagg for HSP70 was determined to be 56.0 ± 0.9 °C (Fig. 2B). Under the experimental conditions used here (non-reducing), SOD-1 appeared as oligomers of > 25 kDa from 45 to 65 °C (Fig. S1) while the unfolded monomerized protein was only detected at > 65 °C (Fig. 2). We therefore could not quantify nor determine a melting temperature for this protein. As expected, total protein levels decreased as temperature increased (Supplementary Fig. S3). This illustrates the suboptimal performance of common loading control techniques, and the superior nature of APP-CTFs, in the CETSA.

To confirm that APP-αCTF protein level remains constant, i.e., soluble across a temperature range, the protein level in both the soluble and insoluble fractions were compared from 4 to 95 °C (Fig. 3). HSP70 was included to illustrate the transition of protein from a soluble to an insoluble form with an increase in temperature, as is expected for most proteins (Fig. 3B). We report that the protein levels of APP-αCTF in the soluble fraction were constant up to 95 °C, with negligible protein detected in the insoluble fraction (Fig. 3A,C). Conversely, as temperature increased, HSP70 level in the soluble fraction inversely reflected its level in the insoluble fraction, clearly demonstrating the movement of protein from a soluble to an insoluble form (Fig. 3B,C). This suggests that APP-αCTF does not aggregate into an insoluble protein at unfavourable temperatures.

**Figure 4:**
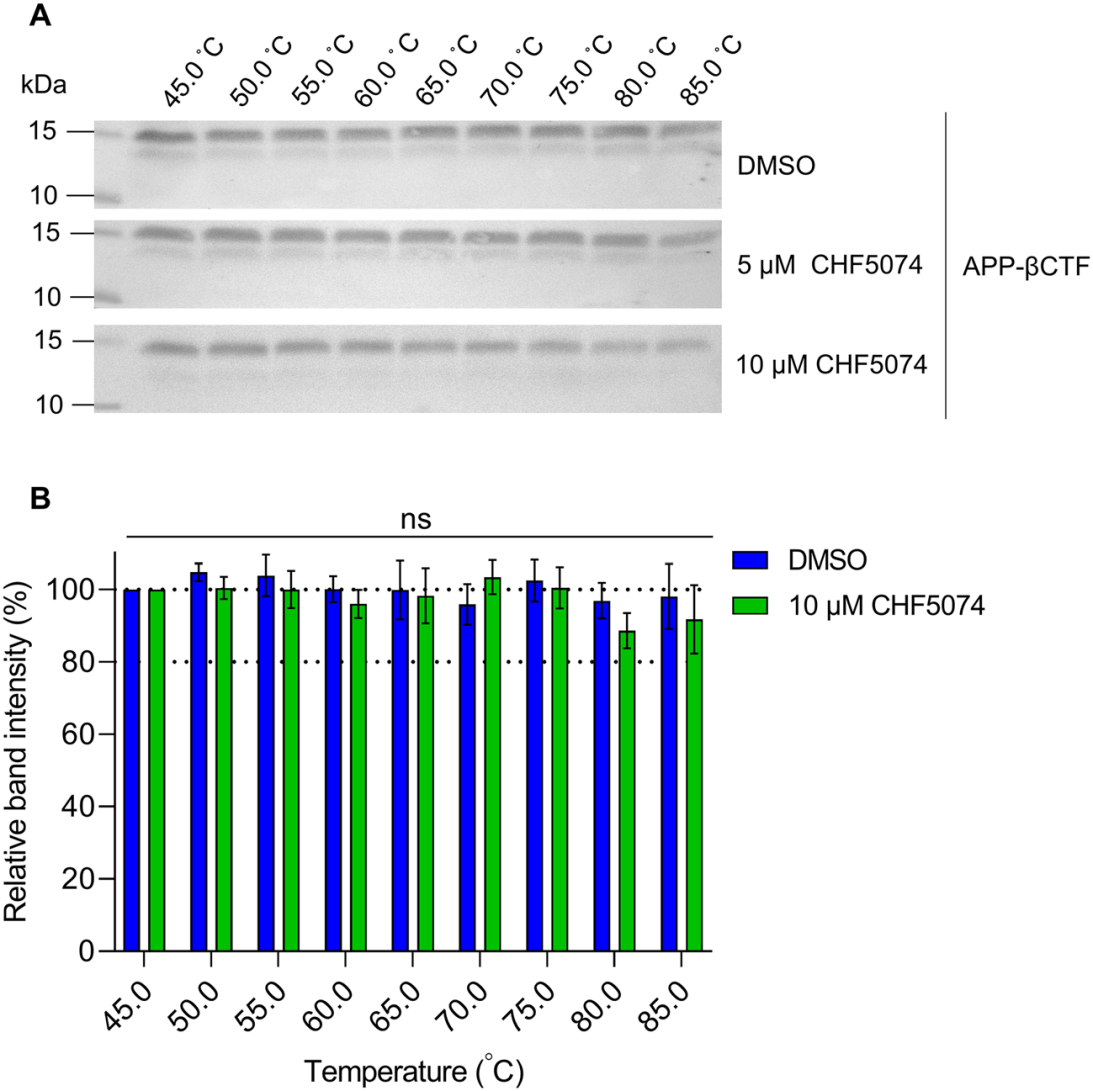
The APP-βCTF binding ligand, CHF5074, has no impact on protein level and stability. (**A**) A representative cropped western blot of APP-βCTF in the soluble fraction from APP-overexpressing HEK293 stable cell line after treatment with 5 and 10 µM CHF5074 for 4 h and subsequent temperature exposure (broad range: 45–85 °C) as compared to a DMSO vehicle control with (**B**) a comparative relative band intensity graph of DMSO (blue) and 10 µM CHF5074 (green) calculated by setting the 45 °C as 100%, as mean ± SD (n = 3). Multiple paired t-test was performed where ns, not significant. See supplementary information file, raw data images for full-length western blots.

We further suspected that APP-CTFs soluble protein levels would remain constant even in the presence of a binding ligand. To assess this, we conducted a broad-range CETSA (45–85 °C) in the presence of the previously identified APP-βCTF binding ligand, CHF5074 (Itanapraced). The assay was conducted in the presence of 5 and 10 µM, concentrations exceeding the reported binding concentration of 3 µM^25^. When our APP-overexpressing cell line—used to generate βCTF—was exposed to CHF5074 and CETSA conducted, the level of βCTF remained constant and comparable to the DMSO control (Fig. 4A). Moreover, the relative band intensity of βCTF in the presence of DMSO and 10 µM CHF5074 were not significantly different across the temperature range tested (*p* > 0.05) (Fig. 4B). This provides evidence that APP-CTF protein level should not fluctuate, even in the event of unexpected off-target binding of ligands tested in this assay. Taken together, this data clearly indicates that APP-CTFs (both α and β) are highly stable soluble proteins with negligible fluctuation in protein level over a broad temperature range (4–95 °C) or in the presence of a binding ligand.

**Figure 5:**
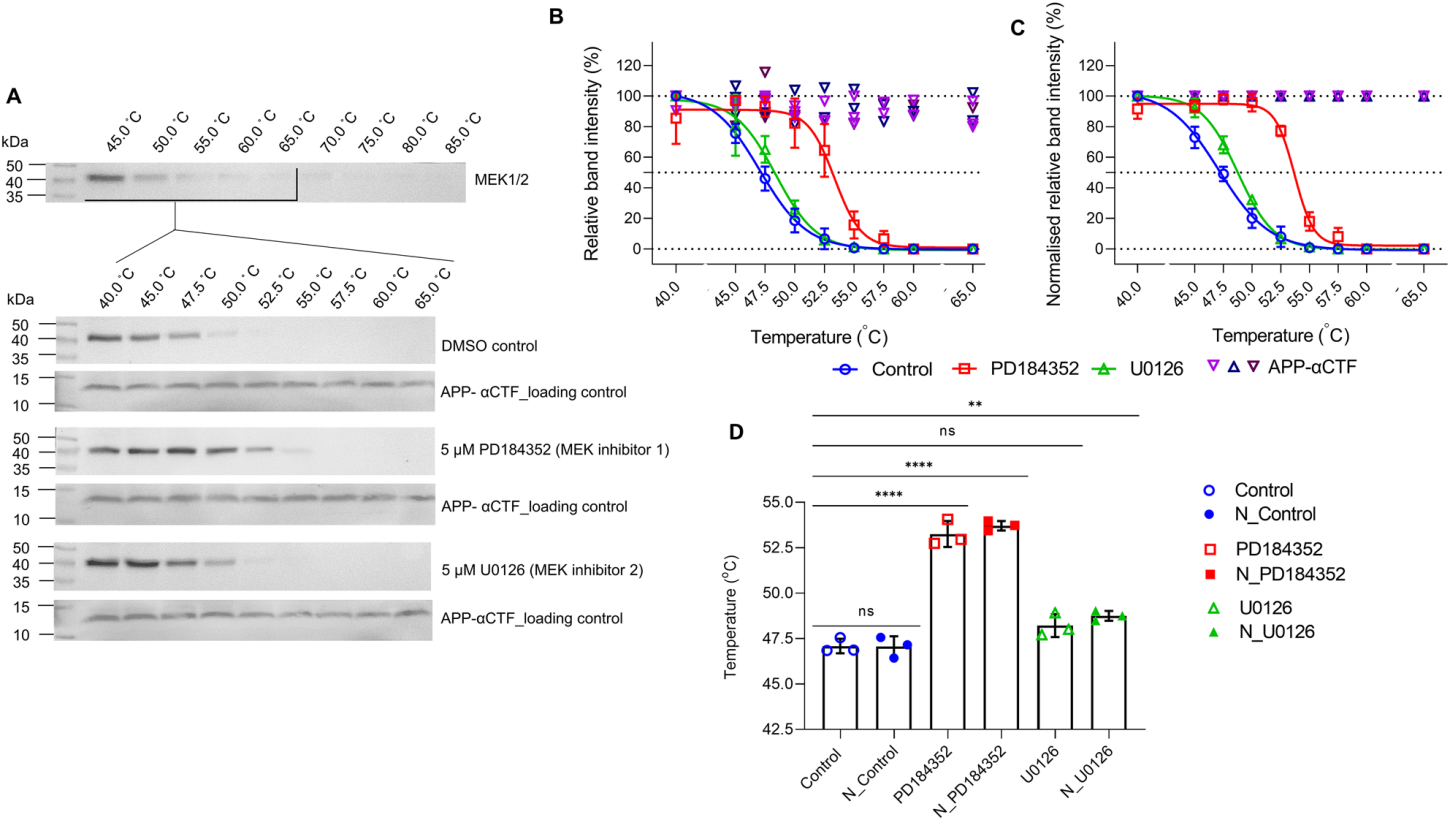
The cellular thermal shift assay of MEK in the presence of inhibitors, PD184352 and U0126 showcasing the use of APP-αCTF as a temperature insensitive loading control. (**A**) A representative cropped western blot of MEK in the soluble fraction from HEK293 cell lysate after treatment with 5 µM inhibitor (PD184352 (open red square) or U0126 (open green triangle)) for 4 h and subsequent temperature exposure (initial broad range 45–85 °C followed by final narrow range 40–65 °C) as compared to a DMSO vehicle control (open blue circle). The corresponding sigmoidal curves (mean ± SD) as determined by relative band intensity (**B**) or normalised (N) relative band intensity, to APP-αCTF loading ([]) (**C**), calculated by setting the highest intensity for each data set as 100% and the curve generated using the Boltzmann sigmoidal equation are shown with (**D**) the mean aggregation temperature (Tagg) ± SD, pre-normalised (open symbol) vs normalised (N—solid symbol) (n = 3). One-way ANOVA with Tukey’s post-hoc was performed where ns, not significant; **p ≤ 0.01; ****p ≤ 0.0001. See supplementary information file, raw data images for full-length western blots.

Next, we ascertained APP-αCTF applicability as a loading control in a CETSA where a protein is successfully stabilised by ligand binding—a working example. We therefore exploited the readily detectable MEK protein in conjunction with two previously identified potent inhibitors, PD184352 and U0126^26^. Based on previous investigations of these inhibitors on the MEK cascade in mammalian cell lines, we determined that 5 µM would be an effective concentration to determine binding^27,28^. As expected, after inhibitor exposure for 4 h, the Tagg of MEK increased in the presence of both PD184352 and U0126 from 47.1 ± 0.4 °C (DMSO control) to 53.4 ± 0.7 °C and 48.2 ± 0.6 °C, respectively (Fig. 5). Of note, the shift obtained with PD184352 was statistically different from the control (**p ≤ 0.0001) while with U0126, it was not (p > 0.05) (Fig. 5D). As previously reported, U0126 does not behave as a tight binding inhibitor which may explain the results observed here^27^. APP-αCTF was clearly detected at all temperatures confirming sample was loaded in each lane (Fig. 5A). The loading control was further used to normalise each relative band intensity and Tagg recalculated (Fig. 5C). In these cases, the normalised (N) Tagg was 47.0 ± 0.6 °C for MEK alone (N_Control), 53.7 ± 0.3 °C in the presence of PD184352 and 48.8 ± 0.3 °C in the presence of U0126 (Fig. 5C,D). Normalised Taggs were not significantly different from the pre-normalised Taggs (*p* > 0.05) however, it was observed that the variance of each data point was reduced in most cases (Fig. 5C,D and Supplementary Fig. S4). Importantly, the increase in Tagg observed for U0126 after normalisation was now found to be significantly different from both pre-normalised and normalised controls (**p ≤ 0.01). This clearly demonstrates the use of APP-αCTF as a genuine loading control for the CETSA where αCTF level remained constant while the protein of interest’s level changed due to stabilisation.

## Discussion

We have identified APP-CTFs, and specifically the αCTF, as temperature stable soluble proteins which can be used as a loading control for the CETSA, with several advantages over other loading controls previously used for this technique. During our study, however, we noted that endogenous APP-αCTF was in low abundance, which necessitated longer exposure times when compared to the other proteins detected. Nevertheless, high-abundance loading control proteins have been criticised as high levels of protein can saturate antibody detection resulting in the inability to perceive changes in protein level (i.e., protein level can appear equal when it exceeds the antibody’s detection range)^29,30^. Alternatives to the use of high-abundance proteins in standard western blots are total protein stains^29–31^, but these cannot be used for the CETSA as total protein follows a general decrease as temperature increases, as confirmed here. We therefore contend that the lower abundance of APP-αCTF can be advantageous as small changes in protein level should be readily detected, even at high total protein concentrations. We also reported how variance in the data was significantly reduced when intensities were normalised—emphasising the capacity to detect changes in loading when using this control.

Importantly, APP and APP-CTFs are extensively studied proteins and therefore numerous antibodies that detect both or just the CTF are available commercially. We did note that the anti-APP_C99 mAb antibody used in our study also detected APP. To avoid interference, it is recommended that antibody incubation be done separately (i.e., the portion of the western blot containing the smaller APP-αCTF should be separated from the rest of the western blot for antibody incubations). The small size of APP-αCTF (detected here at ~ 12 kDa) is also favourable as it allows for protein targets (those > 15 kDa) to be assessed simultaneously with the control, without harsh secondary techniques. Furthermore, the stability observed for APP-αCTF across a broad range of temperatures means a single loading control could be used to assay many different sized proteins thus standardising the CETSA and reducing overall laboratory costs.

Another important, yet unexplored, consideration for loading controls in the CETSA is the possibility of off-target binding during experimental screening of small molecule ligands. This would negatively impact control protein level and downstream data normalisation. Here, we describe that even in the presence of a known binding ligand, CHF5074, the level of APP-βCTF remained constant and therefore, as a loading control, would be unaffected by off-target binding. Since both αCTF and βCTF behave similarly in the CETSA, we suspect that APP-αCTF would be unaffected by off-target binding as well. Since no ligands for αCTF have been identified, this remains to be verified in the future. Conclusively, APP-αCTF is a thermally stable, readily detectable, soluble protein which can be used as a single standard loading control for the CETSA, with superior characteristics when compared to current loading control options.

## Methods

### Cell culture

The HEK293 (ATCC^®^ CRL-1573), HeLa (ATCC^®^ CRM-CCL-2), MDA-MB-231 (ATCC^®^ HTB-26) and A357 (ATCC^®^ CRL-1619) cell lines were maintained at 37 °C with 5% CO_2_ in DMEM supplemented with 10% (v/v) foetal bovine serum (FBS), 0.1× penicillin–streptomycin and 50 µg/ml gentamicin. The SH-SY5Y (ATCC^®^ CRL-2266) cell line was maintained at 37 °C with 5% CO_2_ in DMEM supplemented with 20% (v/v) FBS, 0.1× penicillin–streptomycin and 50 µg/ml gentamicin. The HEK293_APP770 cell line (stable cell line overexpressing APP770)^20^ was maintained at 37 °C with 5% CO_2_ in DMEM supplemented with 10% (v/v) FBS, 0.1× penicillin–streptomycin, 50 µg/ml gentamicin and 900 µg/ml G418.

### Western blot analyses

Each cell line (HEK293, HeLa, MDA-MB-231, A375, SH-SY5Y and HEK293_APP770) was harvested, washed twice with phosphate buffered saline (PBS, 10 mM Na_2_HPO_4_, 1.8 mM KH_2_PO_4_, 137 mM NaCl, 2.7 mM KCl, pH 7.2) and lysed with lysis buffer (RIPA buffer (MilliporeSigma, Massachusetts, USA) supplemented with 1× protease inhibitor cocktail and 1 U DNase) for 1 h at 4 °C with agitation. A protein fraction was obtained by centrifuging at 10,000×*g*, 30 min at 4 °C and supernatant collected. The protein concentration was determined using the Pierce™ BCA protein assay kit following manufacturer’s instructions (Thermo Fisher Scientific, Massachusetts, USA). Each sample was separated by non-reducing SDS-PAGE with Spectra™ Multicolour Broad Range Protein Ladder (Thermo Fisher Scientific, Massachusetts, USA) and transferred to nitrocellulose membrane. Where applicable, western blots were cut horizontally to allow the detection of different proteins within a single experiment or to improve the signal of APP-CTFs by removing the larger full-length APP (also detected with the antibody used here). At least one molecular weight marker was kept above and below the expected protein size, with a minimum of two molecular weight markers per cut section. Individual western blot sections were probed with either mouse anti-APP_C99 mAb (1:1 500) (MilliporeSigma, Massachusetts, USA; cat#MABN380, RRID:AB_2714163), mouse anti-vinculin mAb (1:500) (MilliporeSigma, Massachusetts, USA; cat#V9131, RRID:AB_477629) chicken anti-HSP70 affinity purified IgY (2 µg/ml), rabbit anti-GAPDH (14C10) mAb (1:2 500) (Cell Signalling Technology, Massachusetts, USA; cat#2118, RRID:AB_561053), rabbit anti-SOD-1 (1:750) (EP1727Y, Abcam, UK, cat# ab51254, RRID:AB_882757) or rabbit anti-MEK1/2 (D1A5) mAb (Cell Signalling Technology, Massachusetts, USA; cat#8727, RRID:AB_10829473) followed by donkey anti-mouse IgG, goat anti-rabbit IgG or rabbit anti-chicken IgY secondary antibody conjugated to horse-radish peroxidase (Jackson ImmunoResearch Laboratories, Inc., Pennsylvania, USA; cat#715-035-150, RRID:AB_2340770 or cat#111-035-144, RRID:AB_2307391 or Millipore sigma cat#A9046; RRID:AB_258432 (1:10,000)). The detected bands were visualised using ECL reagent (Novex™ ECL Chemiluminescent Substrate Reagent Kit, Thermo Fisher Scientific, Massachusetts, USA). Amido black total protein stain (0.1% (w/v) amido black in 10% (v/v) acetic acid) was conducted as described by Goldman et al.^31^.

### Cellular thermal shift assay

The CETSA was conducted as described by Jafari et al.^3^ and amended by Delport et al.^6^ and Chambers et al.^32^. For each assay, the cell line (either HEK293 or APP-overexpressing HEK293 stable cell line) was seeded at 1 × 10^5^ cell/ml (in a T75 flask) and grown until 70% confluency (± 3–4 days). For the MEK CETSA, the HEK293 cell line was exposed to either 5 µM of inhibitors, PD184352 (cat#PZ0181) or U0126 (cat#U120, MilliporeSigma, Massachusetts, USA), or an equal volume of DMSO as the control for 4 h. For the APP-βCTF CETSA, the APP-overexpressing HEK293 stable cell line was exposed to either 5 or 10 µM CHF5074 (cat#HY-14399, MedChemExpress, New Jersey, USA), or an equal volume of DMSO as the control for 4 h. The cells were subsequently harvested, collected by centrifugation (500×*g*, 5 min) and washed with PBS twice. The whole cell pellet was resuspended in 900 µl of PBS, aliquoted into 9 equal aliquots of a 100 µl and subjected to a temperature range (broad range: 45–95 °C, narrow range: 40–65 °C in 5, 10 or 15 °C increments) using the ProFlex™ 96-well PCR System (Applied Biosciences™, Thermo Fisher Scientific, Massachusetts, USA) for 6 min, followed by a 6 min cool down period. Samples were also kept at 4 °C and RT. The cell suspension was centrifuged (500×*g*, 5 min) and the collected pellet lysed with lysis buffer for 15 min at 4 °C, using an end-over-end mixer. The samples were centrifuged (10,000×*g*, 30 min) to obtain a supernatant containing soluble protein and a pellet containing insoluble aggregated protein. The soluble protein fraction was used as is for western blot analyses (under non-reducing conditions as described above). The insoluble protein fraction was resuspended in an equal volume of PBS and sonicated (3 × 5 s, 10 s rest, on ice) prior to analysis by western blot (under non-reducing conditions as described above).

### Quantification and statistical analysis

All band intensities were determined using ImageJ^33^. For total protein stain analysis, six regions were selected horizontally across the blot and densitometry performed from each area, in each lane. These were subsequently added to obtain a sum of total protein intensity for each lane. For antibody detected band analysis, densitometry with background excluded was determined for each band. Standardized optical density was determined by dividing each band intensity by the mean of all intensities for each blot. Relative band intensity was determined by setting the highest value to 100% in each case. All experiments were repeated a minimum of three times and all statistical analyses performed in GraphPad Prism 9 (2021). Replicates are indicated in each figure legend where each n is a repeat of the entire experiment. The simple linear regression equation was used to determine the relationship between protein concentration and protein intensity. All relative CETSA data was analysed by non-linear regression using the Boltzmann sigmoid equation as described by Jafari et al.^3^ to determine Tagg. For corrected MEK CETSA, the densitometry for MEK and for APP-αCTF was determined for each temperature. A correction factor was determined for each lane by dividing the band intensity of APP-αCTF in the first lane by the band intensity for APP-αCTF in each lane. This correction factor was then multiplied by the densitometry of MEK and APP-αCTF in each lane. Relative band intensity was calculated (i.e. APP-αCTF will be at 100% for each lane) and used to determined Tagg as described above. One-way ANOVA with Tukey’s post hoc or multiple paired t-test was used to determined statistical differences where applicable (α = 0.05, 95% CI).

### Equipment and settings

All images were obtained using the G:BOX Chemi XR5 (Syngene, India) in the GeneSys software (2012). Series mode was selected for imaging, followed by ELC reagent with visible marker as the detection method where two images were obtained with no binning. Western blots detecting APP-CTFs and SOD-1 were exposed for 2 × 5–10 min (10–20 min total) for best results. The exposure for all other proteins (vinculin, HSP70, GAPDH, MEK and β-actin) was done for 2 × 1–2 min (2–4 min total). Merged western blot images with molecular weight markers were obtained after exposure. White light molecular weight marker images were brightened to allow for better resolution of the marker in the final merged image, in some cases. No excessive manipulation nor processing was done on any of the images and all final images represent the original data.

## Supplementary Information

Supplementary Figures.

## Data Availability

All data generated and analysed during this study are included in this published article and as a Supplementary Information File.
