# Supplementary material for: A superior loading control for the cellular thermal shift assay
Source: Sci Rep. 2022 Apr 23;12:6672. doi: 10.1038/s41598-022-10653-7 (PMC9035151; doi:10.1038/s41598-022-10653-7)

Title page

Title: A superior loading control for the cellular thermal shift assay

Author Affiliation: Alexandré Delport*[,a], Raymond Hewer[a].

[a]Discipline of Biochemistry, School of Life Sciences, University of KwaZulu-Natal, Pietermaritzburg, South Africa, 3201.

ORCID: Alexandré Delport: 0000-0003-3511-4369, Raymond Hewer: 0000-0002-6025-8826.

Corresponding Author: Dr Alexandré Delport*

Telephone number: +27 33 260 5150
Email: delporta1@ukzn.ac.za

Keywords: CETSA, loading control, thermal stability, normalisation, amyloid precursor protein C-terminal fragments (αCTF/βCTF)

Supplementary Material

Figure S1: The cellular thermal stability of β-actin and SOD-1. A) A representative western blot of β-actin in the soluble fraction from HEK293 cell lysate after temperature exposure (4 °C, RT, 45-65 °C) with B) corresponding relative band intensity sigmoidal curve, as mean ± SD, calculated by setting the highest and lowest intensity as 100 % and 0 %, respectively and the curve generated using the Boltzmann equation (n = 5). C) A representative western blot of SOD-1 in the soluble fraction from HEK293 cell lysate after temperature exposure (45-85 °C) showing detection of high molecular weight oligomers. RT, room temperature (Figure 2, main text). See raw data images for full-length western blots.

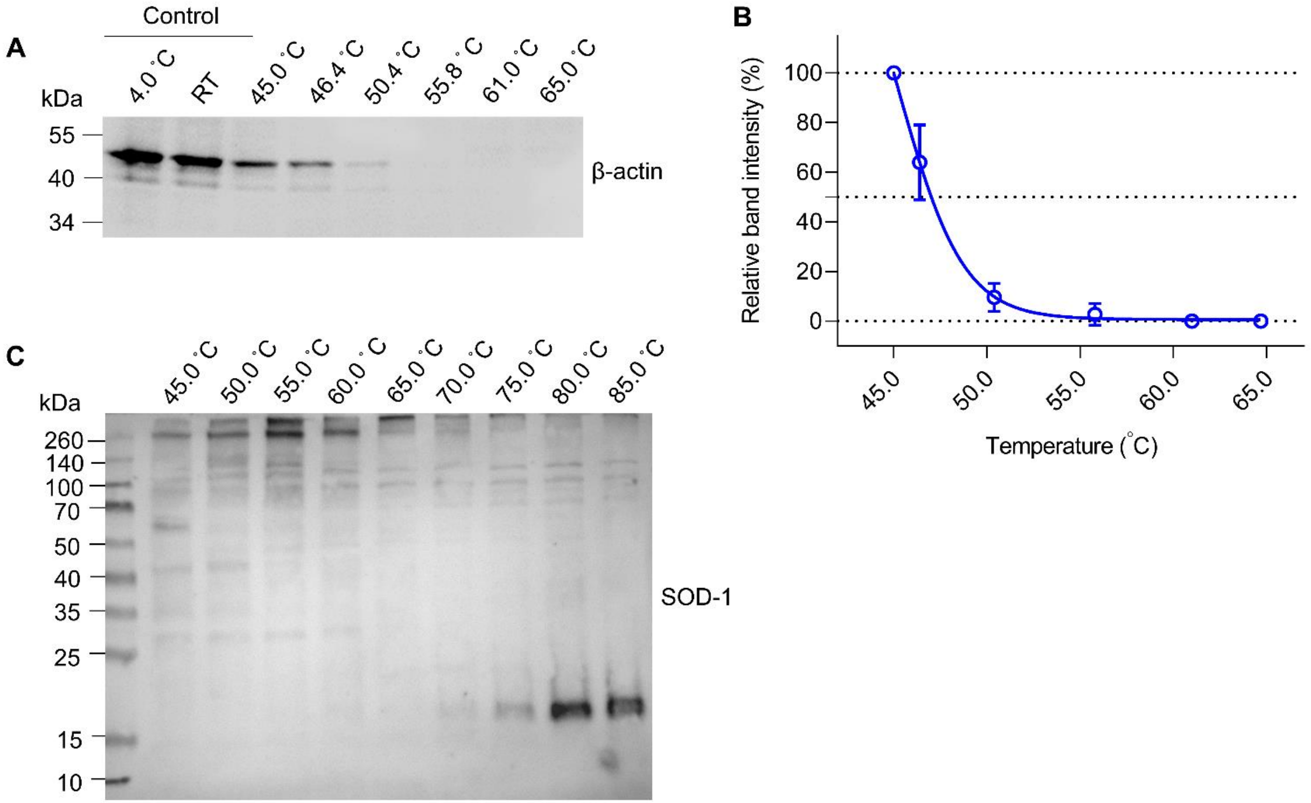

Figure S2: The cellular thermal stability of APP-βCTF. A) A representative western blot of the soluble fraction from APP-overexpressing HEK293 cell lysate after temperature exposure (45-85 °C) of APP-CTFs, βCTF and αCTF with B) corresponding relative band intensity graph calculated by setting the 45 °C as 100 % with mean ± SD, where βCTF shows temperature insensitivity comparable with αCTF using a multiple paired t-test where ns, not significant (Figure 2 and 3, main text). See raw data images for full-length western blots.

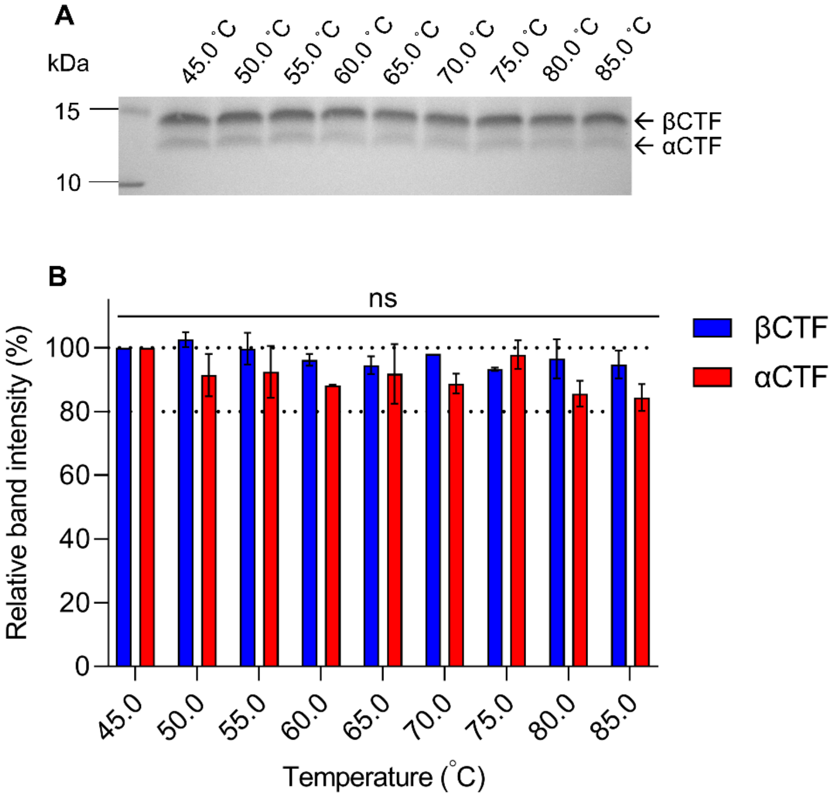

Figure S3: Total protein cellular thermal stability. A) A representative western blot of the soluble fraction from HEK293 cell lysate after temperature exposure stained with an Amido black total protein stain with B) corresponding relative band intensity curve as mean ± SD (n = 6). See raw data images for full-length western blots.

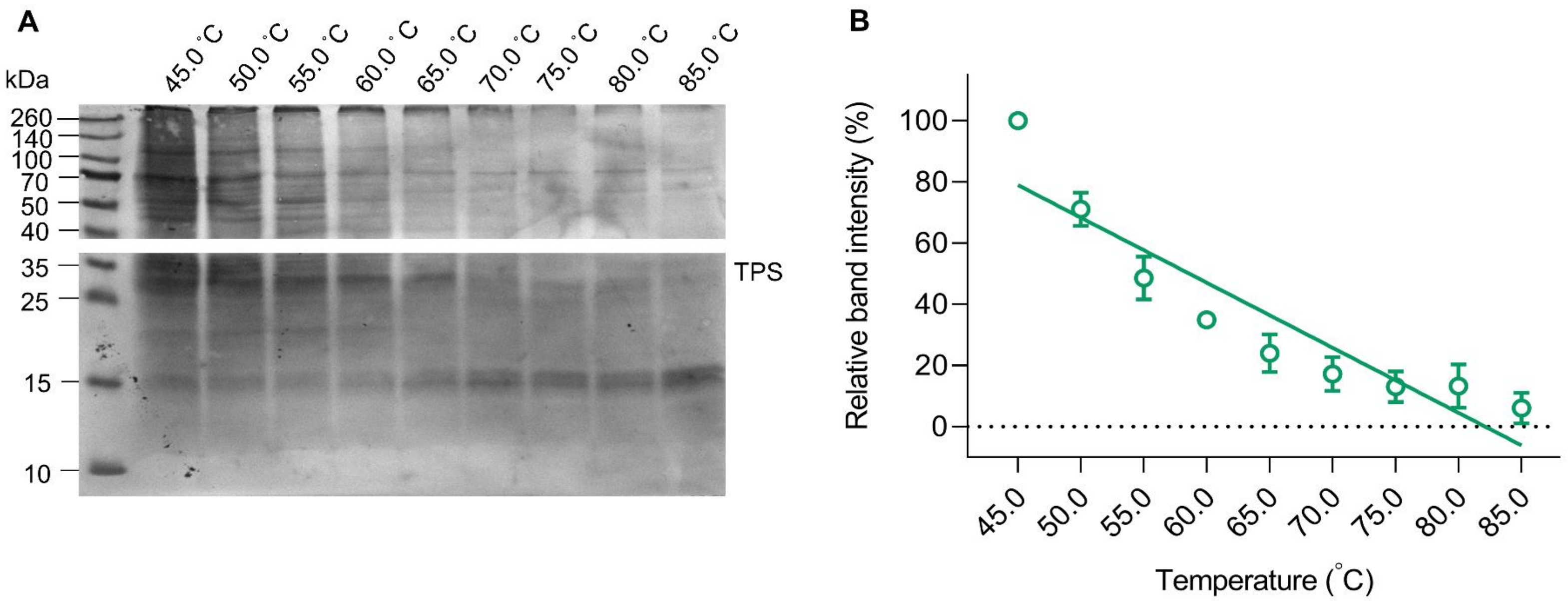

Figure S4: Temperature data variance for the MEK CETSA. Pre-normalised (solid line) or normalised (N-dotted red line) relative band intensity of individual data points at specified temperatures of MEK from the soluble fraction from HEK293 cell lysate in the absence (DMSO control – A) or presence of inhibitors, PD184352 (B) or U0126 (C) displaying data variability.

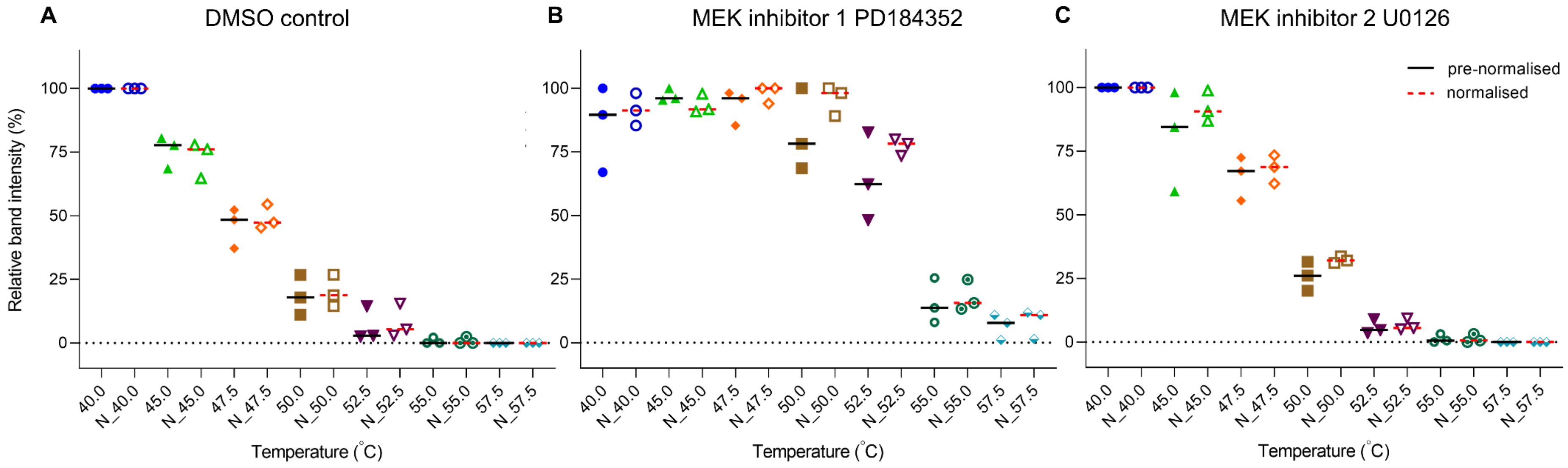

Raw Data Images

Of figure 1 in the main text: Western blot from Figure 1A was imaged as a full-length western blot. After which the western blot was cut above the 15 kDa marker and imaged again. APP-CTF signal was found to be improved when full-length APP sections were removed as conveyed in the discussion.

Figure 1A_Concentration range

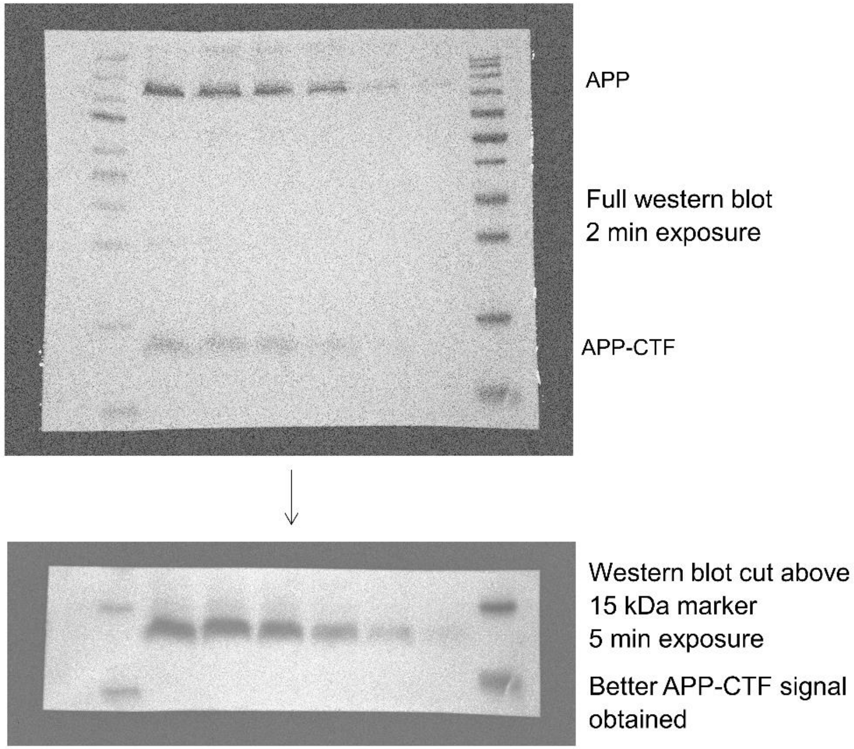

Figure 1A_TPS densitometry regions

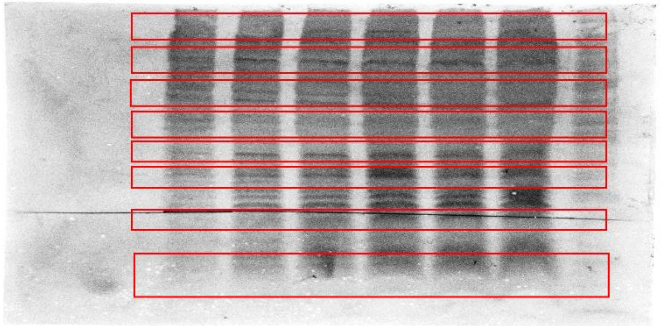

Figure 1B_Mammalian cell lines

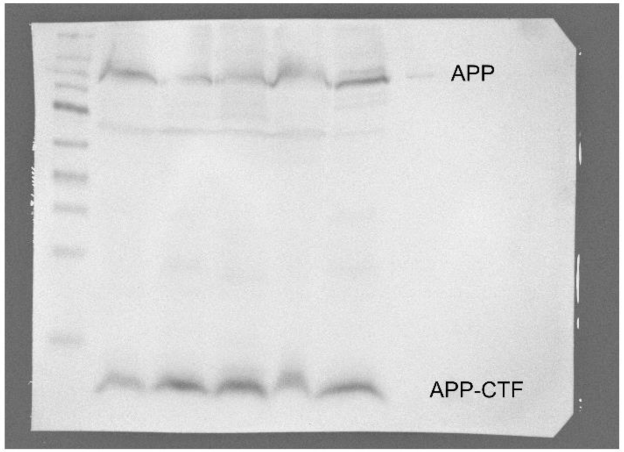

Of figure 2 in the main text: Each western blot was cut prior to antibody hybridisation. The sections showing HSP70 and GAPDH were imaged simultaneously.

Figure 2A

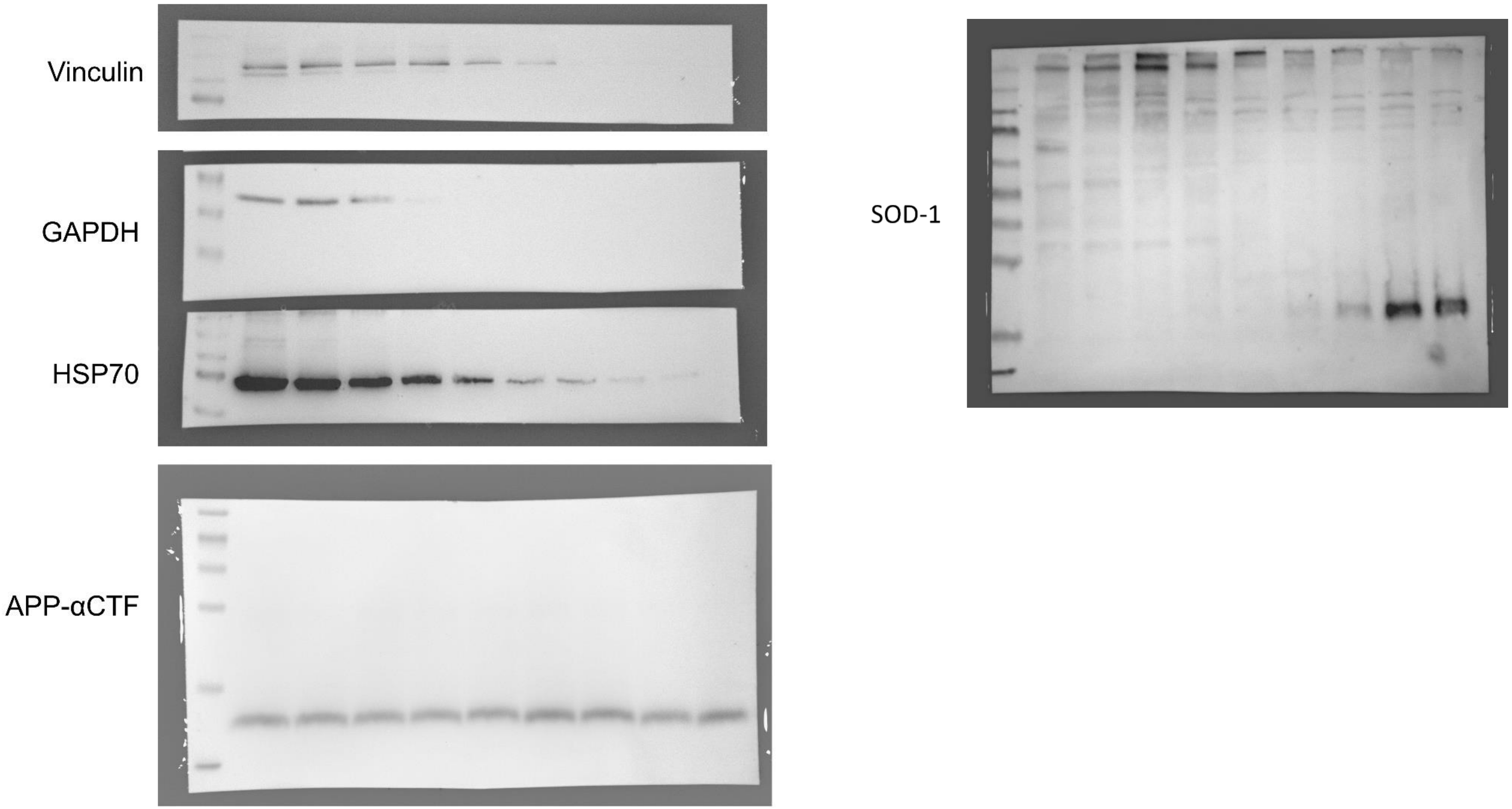

Of figure 3 in the main text: Each western blot was cut prior to antibody hybridisation where each HSP70 and APP-αCTF sections are from a single blot.

Figure 3A and B

APP- αCTF and HSP70_4 °C, RT, 40-95 °C

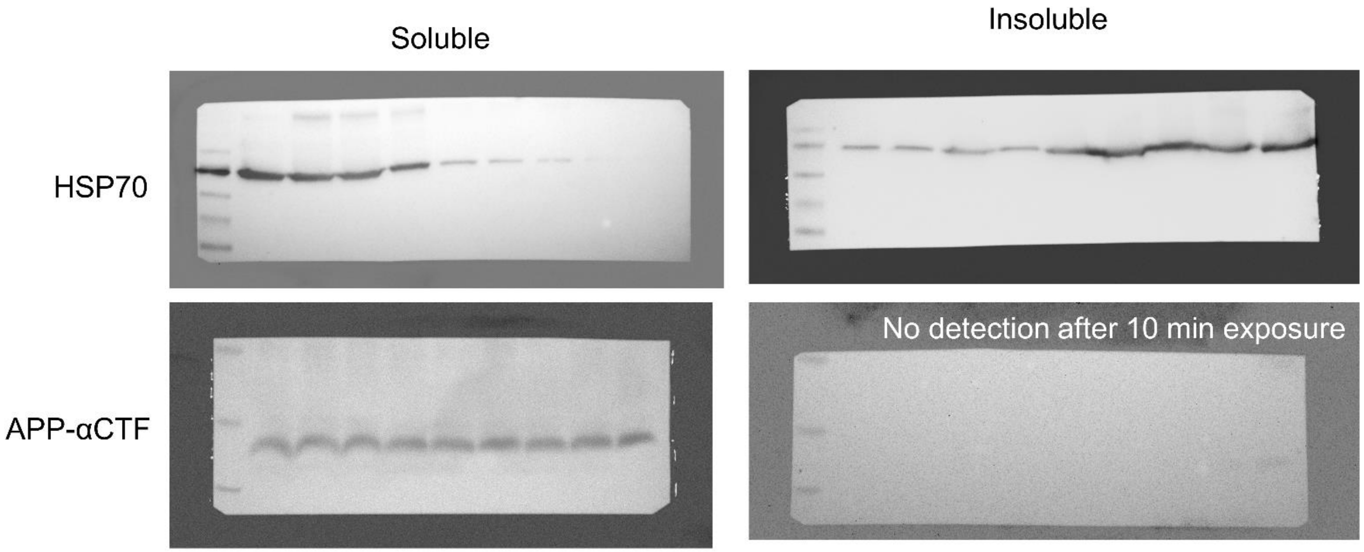

Of figure 4 in main text: Western blots were cut prior to antibody hybridisation (APP-CTF signal was found to be improved when full-length APP sections were removed as conveyed in the discussion).

APP-βCTF + DMSO

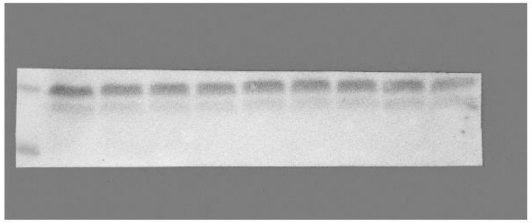

APP-βCTF + 5 μM CHF5074

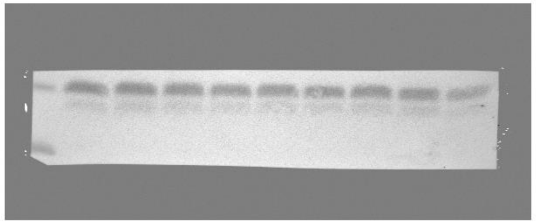

APP-βCTF + 10 μM CHF5074

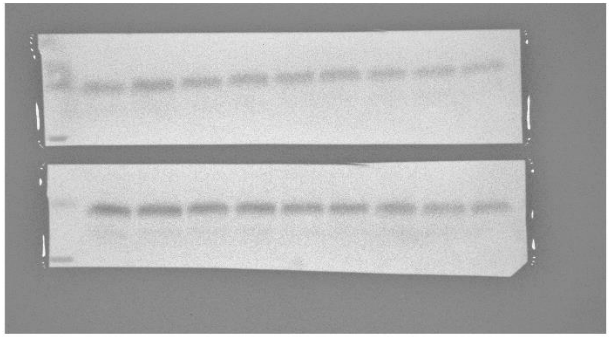

Of figure 5 in the main text: Western blots were cut prior to antibody hybridisation. Western blot sections showing MEK_40-65 °C (3) and APP-αCTF (3) were imaged simultaneously. Each APP-αCTF section removed from corresponding MEK_40-65 °C section.

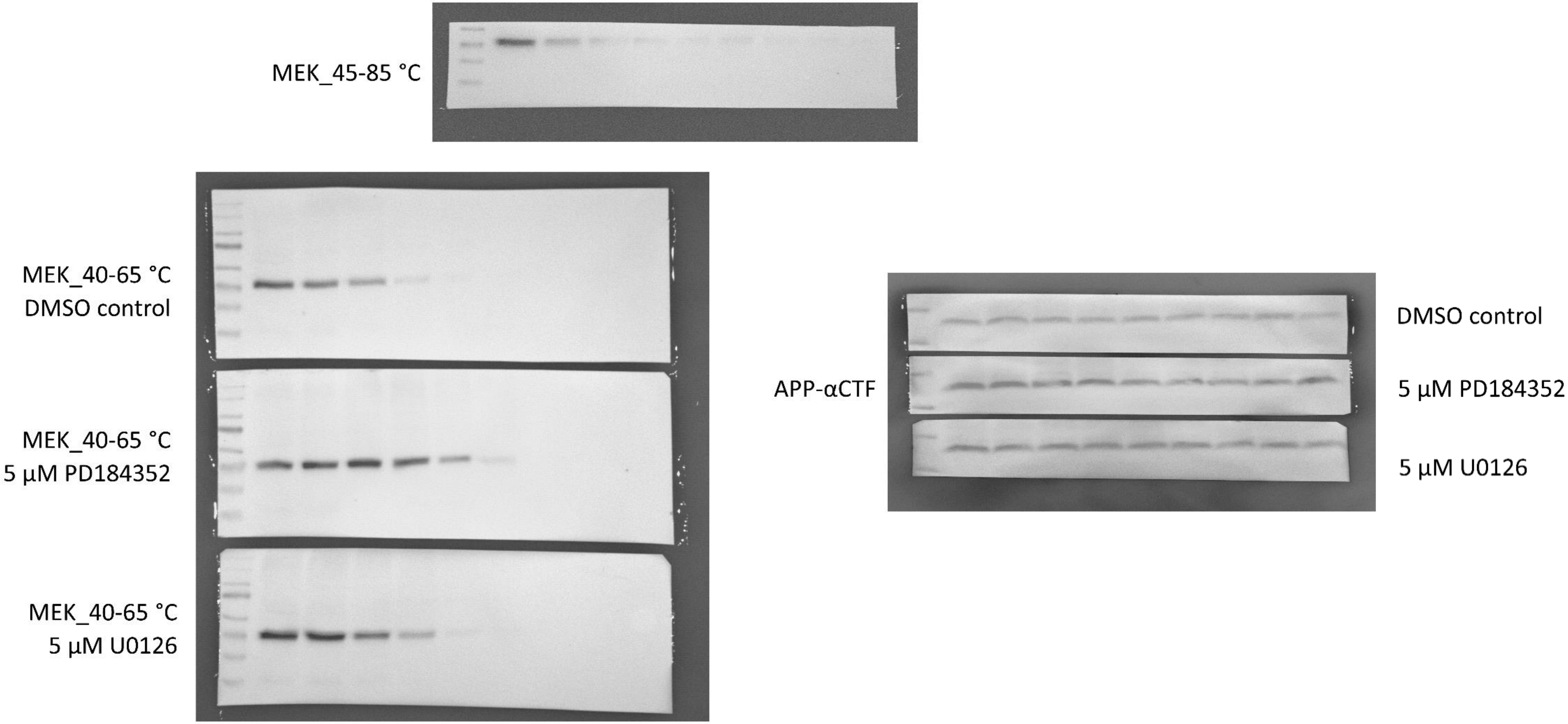

Of figure S1 in the supplementary data:

Figure S1

β-actin_4 °C, RT, 45-65 °C

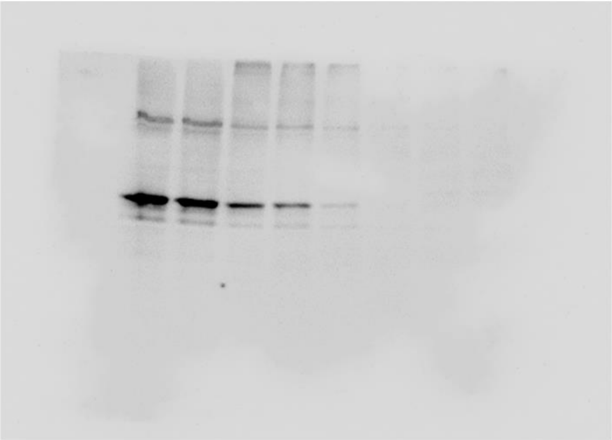

Of figure S2 in the supplementary data: Western blots were cut prior to antibody hybridisation (APP-CTF signal was found to be improved when full-length APP sections were removed as conveyed in the discussion).

APP-βCTF and -αCTF

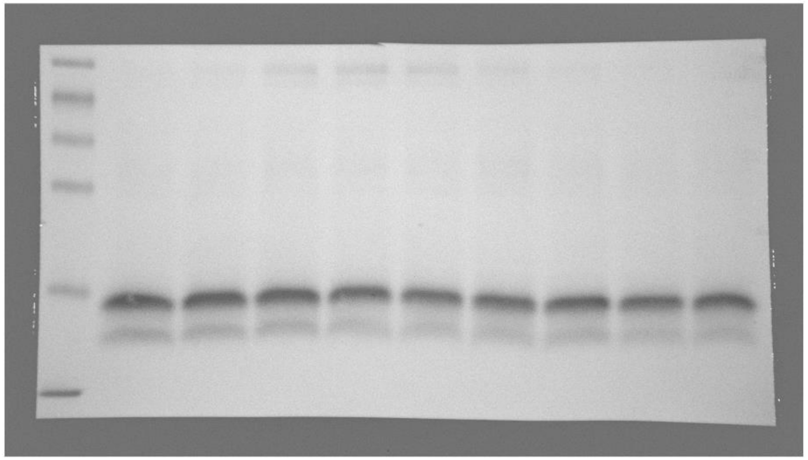

Of figure S3 in the supplementary data:

Figure S2_TPS densitometry regions

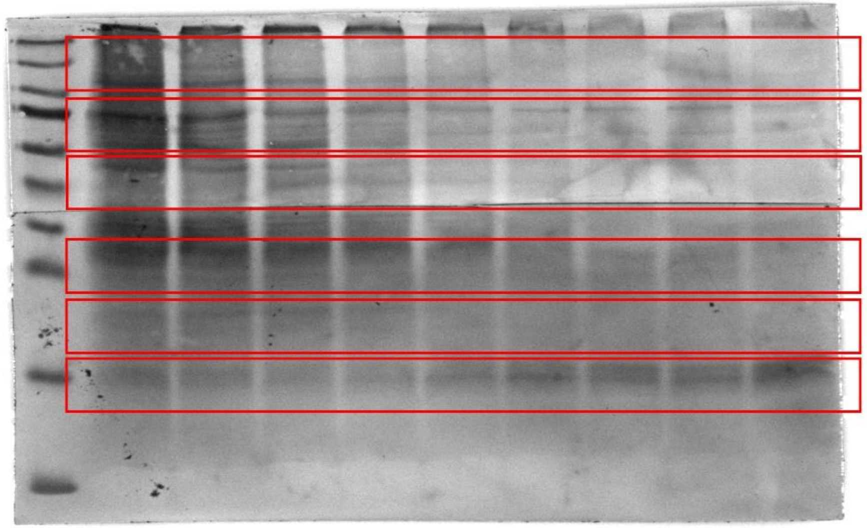

Supplement: Supplementary file 1 — Supplementary Figures. [file 41598_2022_10653_MOESM1_ESM.pdf]
